# The Dynamicity of the Oxytocin Receptor in the Brain May Trigger Sensory Deficits in Autism Spectrum Disorder

**DOI:** 10.3390/cimb47010061

**Published:** 2025-01-17

**Authors:** Claudia Camerino

**Affiliations:** 1 Department of Precision and Regenerative Medicine, School of Medicine, University of Bari Aldo Moro, P.za G. Cesare 11, 70100 Bari, Italy; ccamerino@libero.it; 2 Department of Physiology and Pharmacology “V. Erspamer”, Sapienza University of Rome, P.le Aldo Moro 5, 00185 Rome, Italy

**Keywords:** oxytocin, oxytocin receptor, thermoregulation, autism spectrum disorder, sensory processing, skeletal muscle, Diagnostic and Statistical Manual of Mental Disorders (DSM-5)

## Abstract

Sensory processing abnormalities have been noted since the first clinical description of autism in 1940. However, it was not until the release of the fifth edition of the Diagnostic and Statistical Manual of Mental Disorders (DSM-5) in 2013 that sensory challenges were considered as symptoms of autism spectrum disorder (ASD). Multisensory processing is of paramount importance in building a perceptual and cognitive representation of reality. For this reason, deficits in multisensory integration may be a characteristic of ASD. The neurohormone oxytocin (Oxt) is involved in the etiology of ASD, and there are several ongoing clinical trials regarding Oxt administration in ASD patients. Recent studies indicate that Oxt triggers muscle contraction modulating thermogenesis, while abnormal thermoregulation results in sensory deficits, as in ASD. Activation of the Oxt system through exposure to cold stress regulates the expression of oxytocin receptor (Oxtr) in the brain and circulating Oxt, and if this mechanism is pathologically disrupted, it can lead to sensory processing abnormalities since Oxt acts as a master gene that regulates thermogenesis. This review will describe the sensory deficits characteristic of ASD together with the recent theories regarding how the modulation of Oxt/Oxtr in the brain influences sensory processing in ASD.

## 1. Introduction

Autism is a complex neurodevelopmental condition and its cause is still unknown. ASD is characterized by two main symptoms: persistent social communication and interaction difficulties and restrictive and repetitive behavior, interests and activities [1]. The majority of research on autism spectrum disorder (ASD) has focused on social, communication and learning/cognitive challenges associated with the disease [2]. However, it is only in the last 10 years that a new diagnostic criterion for ASD has been accepted by the scientific community and included in the Diagnostic and Statistical Manual of Mental Disorders (DSM-5): sensory processing [3]. Indeed, abnormalities in sensory processing can influence behavioral and cognitive experience in ASD patients since alterations in social cognition are marked by a very different perceptual experience of the world. Atypical sensory experience is estimated in about 90% of ASD individuals in every sensory modality, such as taste, touch, audition, smell and vision. The neurobiological alterations that affect processes as diverse as cognition and sensory experience in ASD and the common thread between them are still unclear. In order to begin to understand the disease, it is essential to clarify whether sensory difference represents secondary consequences after reduced social interaction or sensory deficits influence both development and neurobiology. ASD affects human experience from sensation to perception, to motor behavior and cognition. This is why it is important to understand how these diverse domains can be related. Research on sensory symptoms may help in simplifying this complexity by focusing on circuit-level alterations in the brain that may affect cortical processing in ASD and offer translational and pharmacological potential to cure this disease. Indeed, according to the “sensory-first” theory, social/cognitive symptoms may be downstream effects of atypical sensory processing in early development, while according to the “top-down” theory, symptoms relating to sensation and social cognition might co-arise from alteration in attention or prolonged lack of social interaction [2,3]. Perturbations in the inhibitory neurotransmitter gamma-aminobutyric acid (GABA) receptor have been associated with autism, and GABAergic signaling is disrupted in several different mouse models of autism [2,3]. Other neuromodulators such as testosterone and oxytocin (Oxt) modulate GABAergic signaling and are associated with autistic traits [4,5,6,7,8]. Oxt is a neurohypophysial hormone, as previously described [9]. Oxt triggers skeletal muscle contraction through thermoregulation, and cold stress exposure increases oxytocin receptor (Oxtr) expression in the brain and the Soleus muscle (Sol), while it decreases circulating Oxt in mice, leading to what we have called “The oxytonic effect” [10]. Oxt’s regulation of thermogenesis is linked to Prader–Willi syndrome (PWS) and Schaaf–Yang syndrome (SYS) since both these pathologies present increased circulating Oxt levels, muscle hypotonicity, decreased Oxtr expression in the brain and frequent episodes of hypothermia and hyperpyrexia with no infection [11,12]. PWS and SYS patients also present a very high incidence of ASD [13] and they often present a history of low body temperature and/or high body temperature with no apparent reason provided on their medical history form [12]. PWS arises from the lack of expression of paternally inherited genes on chromosome 15q11-q13 or maternal uniparental disomy or an imprinting defect. Mage family member L2 (Magel2) is one of the affected genes located on 15q11-q13 and mutations in Magel2 have been found in PWS, SYS and ASD individuals [11,12]. In a mouse model of autism in which Magel2 has been knocked down, Oxtr is downregulated or upregulated according to postnatal Oxt treatment [14]. Magel2-KO mice present neurodevelopmental impairment and autistic-like behavior [12]. In this review, we explain the sensory deficits described in ASD in light of the novel theories that see Oxt/Oxtr expression as being dynamically modulated in the brain, and we hope that these results may be instrumental in designing precisely timed Oxt-based therapeutic strategies that target specific brain regions and can improve the clinical features, both sensorial and social, of ASD.

## 2. Autism and Sensory Deficits

In this section, we will describe some of the sensory deficits documented in ASD and their manifestations (Table 1). Pediatric patients with ASD suffer hyper- or hyposensitivity to visual, auditory, tactile and olfactory stimuli. ASD individuals also report paradoxical heat sensations when cold is perceived as burning hot, indicating disruption of thermosensory integration since central processing of information seems to be altered in ASD rather than peripheral perceptions [15,16]. The existence of sensory abnormalities in ASD was only recently included in the DSM-5 as diagnostic criteria for ASD (American Psychiatry Association 2013) and have for a long time been overlooked [15]. Central mechanisms during the integration of multisensory stimulus rather than peripheral mechanisms cause sensory deficits in ASD. In the DSM-5, both hyper- and hypo-reactivity to sensory input or unusual interest in sensory aspects or the external milieu are mentioned as diagnostic criteria for ASD. These criteria include somatosensory abnormalities such as indifference to pain related to temperature, adverse response to texture or excessive touching of objects (APA 2013). For example, vibro-tactile stimulus perceived as static and poor vibro-tactile amplitude discrimination have been reported in children with ASD [16]. Hypersensitivity to thermal pain and innocuous thermal stimuli has been found in ASD in adults [17]. Conversely, adolescents with ASD showed normal detection of thermal pain but hyposensitivity to innocuous thermal stimuli [15]. Moreover, in adolescents and adults with ASD, normal pain detection was observed during thermal and electrical stimulation [18], where pressure pain was also lower in children [19]. In a recent study, the somatosensory perception of patients with ASD was investigated, since altered detection of somatosensory stimuli may cause unusual sensory perception in ASD using the quantitative sensory testing (QST) protocol on neuropathic pain. Hyper- or hyposensitivity to sensory inputs is normal in certain parts of the body and abnormal in other parts, in line with the different innervation from the many brain areas with peripheral and central neuronal activity. ASD patients show similar thresholds in detecting innocuous warmth and cool and light pressure on their palm and forearm, but hypersensitivity to vibrations was seen in ASD on the forearm and hypersensitivity to thermal pain at both sites. Interestingly, individuals with ASD exhibit enhanced perception of certain stimulus properties such as vision, where local, circumscribed properties of a visual stimulus are seen instead of the complete picture. This peculiarity of ASD is described as follows: “ASD individuals see the tree but not the forest”. A similar pattern of perception is also present in the auditory domain where the change of a single note of a melody is perceived to be stronger than global melodic changes [20]. That sensory deficits in ASD vary according to the age of patients and the area of the body is an important concept consistent with the plasticity of the Oxtr expression in the brain, as we will explain in the following sections [13].

## 3. Oxytocin and Sensory Deficits in Autism Spectrum Disorder

Oxt/Oxtr^−/−^ mice have impaired thermoregulation, resulting in thermosensory deficits and the inability to maintain a stable temperature [21,22]. Our previous studies indicate that temperature regulation is impaired in PWS individuals since they show significant changes in thermoregulation compared to siblings or controls [19,21,22,23]. PWS is caused by the loss of expression of a critical genetic region on chromosome 15q11-q13 and a very high percent of PWS individuals also present ASD. Our studies also indicate that Oxt acts as a master gene regulating thermogenesis and influences the manifestation of the PWS phenotype [9,13]. A small but statistically significant proportion of PWS children have been reported to experience persistent or episodic hyperpyrexia/hypothermia [11], whereas fingertip temperature is unaltered [22,24,25]. About 90% of ASD individuals have a sensory abnormality that manifests with hyper- and hypo-reactivity to smell, taste, audition, vision and tactile sensitivity. The sensory deficit influences the perception of the external word and social behavior [26]. Oxt is also released in response to tactile stimuli, and Oxt in the plasma of autistic children is decreased compared to neurotypical individuals [27]. Abnormalities in the gene that encodes for Oxtr [28] as well as in Oxt have been found in ASD [29]. This is why we hypothesize that Oxt regulation of thermogenesis can cause sensory abnormalities in ASD (Table 2). Moreover, children with ASD present altered thermal thresholds with reduced sensitivity to warmth, coolness and heat, but not pain threshold. We speculated that this evidence may be significant since ASD individuals, unlike PWS individuals, do not have hypothalamic syndrome, which can per se cause temperature fluctuations and sensory abnormalities. However, a more comprehensive study of ASD is not within the scope of this article, which regards exclusively the relationship between ASD and sensory deficits. Oxt’s regulation of thermogenesis may be responsible for sensorial functionality and body temperature regulation through muscle contraction in healthy individuals, while a dysfunctional Oxt system can cause varying degrees of sensory abnormalities and muscle hypotonicity, as seen in PWS and ASD.

## 4. Oxytocin Receptor Modulation Is Dynamic Across Lifespan and Is Sexually Dimorphic

Oxt levels are lower in autistic compared with neurotypical children; however, this difference is not detectable in adults [30]. ASD is characterized by deficits in social interaction and communication and restricted interests together with sensory deficits (APA 2013). These traits are all thought to be mediated by Oxt. Indeed, several components of the Oxt/Oxtr system have been associated with ASD. Variations in these genes can affect Oxt/Oxtr expression and distribution. Some studies found Oxt levels to be inversely correlated with ASD severity; however, the expression of Oxtr in the brain has not been investigated. The fact that Oxt is lower in autistic children highlights an involvement of the Oxt system in the development or manifestation of ASD [28]. From this perspective, the Oxt system can shape the brain for social interaction and sensory perception during a critical period in infancy during which there is probably a critical window when Oxt administration may be effective. However, little is known about the developmental trajectory of Oxt/Oxtr and the causality of these manifestations in ASD, which is why studies on the genetics of Oxt/Oxtr are necessary. In this regard, thermoregulation may modulate Oxtr in the brain and cause sensory deficits in ASD [30]. The experimental manipulation of Oxtr expression levels and Oxtr gene KO can have a significative effect on behavior and physiology [31]. This can lead to the identification of a critical window of Oxtr expression in the brain together with associated genes, which is important for understanding ASD and other psychiatric illness. Oxt/Oxtr expression is dynamic throughout a person’s development, with critical periods. Indeed, Oxtr peaks in early childhood and late adulthood and is highly correlated with dopaminergic signaling across a lifespan to adapt to shifting environmental challenges. The peak in Oxtr expression observed during early childhood is probably stronger in males because it is influenced by gonadal steroids. These sex differences may contribute to the reported sex differences in neurodevelopmental disorder diagnoses and is consistent with the sex bias of ASD recurring more often in males than females. Specifically, the early childhood peak of Oxtr binding that was observed in brain tissue of the ventral pallidum tissue from neurotypical donors was absent in the same tissue in autistic donors. Moreover, Oxtr expression and binding are higher in the ventral pallidum and nucleus basalis of Meynert brain tissue of neurotypical donors than the same tissue of autistic donors [31]. Oxtr undergoes epigenetic modifications, and these modifications predict the severity of symptoms in adults with ASD. Indeed, the neural networks involved in reward processing and social capability in ASD involve the Oxt/Oxtr system, which is sensitive to epigenetic processes caused by environmental exposure, and these epigenetic modifications account for the features of autistic traits. Specifically, Oxtr hypermethylation in the intron 1 area of MT2 was related to a less severe developmental phenotype making this site a potential biomarker of adults with ASD with less severe verbal communication deficits [32]. It is worth noting that thermoregulation and thermogenesis can also cause epigenetic modifications of Oxtr.

## 5. Current Theories Explaining Sensory Deficits in Autism Spectrum Disorder

Individuals with ASD “See the trees but not the forest”, which means that ASD individuals see the details of the perceptual world rather than the global picture [30]. To understand autistic sensory experience, the perceptual processing cannot be simply characterized as a talent or a deficit reflecting neither hyposensitivity nor hypersensitivity, but sensory experience in ASD exhibits a bias toward local versus global characteristics of a sensory scene which can be more or less advantageous according to the task [2]. Moreover, these sensory processing abnormalities also impact other domains of ASD such as social communications, worsening their severity [3]. This is why individuals with ASD have enhanced performance in tasks that rely on the analysis of stimulus details but have difficulties when these details need to be integrated into a complete image. Sensory difference is a common issue in ASD and much of our cognitive and social representations are dependent upon sensory inputs. In this regard, there are at least five theories that explain the ASD. The first theory, also called “Theory of mind” [33], suggests that ASD individuals have a decreased ability to understand other people’s feelings and this can be caused by abnormalities in sensory processing. Neuroimaging studies reveal that inferior frontal regions including the mirror neuron system and the temporoparietal junction are involved in these tasks. The second theory, named “Weak central coherence” [34], proposes that the meaning of things is built through the integration of information across lower-level sensory and higher-level cognitive processing, and this is abnormal in ASD individuals. The classic illustration of how this theory works is that to understand a complex visual scene, the integration of all details is necessary and not focusing on single details, as seen in the tree and forest example. The third theory is named “Predictive coding hypothesis” and is based on the notion that individuals with ASD do not have a robust historical representation of the world, making it difficult for them to predict upcoming events and limiting interactions with external environments. This explains, for example, the tendency of ASD individuals to engage in repetitive behavior to limit novel sensory inputs in an endlessly novel world [35]. The fourth theory is called “Reduced sensory precision and reliability” and states that changes in the variability of neural response patterns create variability (or less reliability) in their behaviors and perceptions [36]. Finally, the fifth and last theory is based on evidence that GABA functioning is altered in ASD, leading to increased excitation for greater glutamatergic signaling, while inhibition is decreased for less GABAergic signaling. Indeed, glutamate and GABA signaling are involved in cortical function, including sensory processing and social function. In this context, increased excitability may explain hypersensitivity in sensory processing, with sensory input eliciting an abnormally large cortical response that makes the experience overwhelming [4,5,6,7,9,36]. This theory has been confirmed by a recent study on animal models [37]. However, here, we propose a novel theory according to which the sensory deficits in ASD can be triggered by the differential modulation of Oxtr in the brain, as we explain in the following sections.

## 6. Oxytocin and Oxytocin Receptor Expression Modulation in the Brain May Trigger Sensory Deficits in Autism Spectrum Disorder: New Theories

In this section, we will describe the most recent studies regarding the modulation of Oxtr in the brain and how it can impact sensory deficits in ASD. In particular, we will focus on four recent studies focusing on the Oxt/Oxtr system.

### 6.1. Oxytocin Is a Master Gene That Regulates Thermogenesis, and Cold Stress Modulates Oxytocin Receptor in the Brain and Peripheral Oxytocin

The first study [10] shows that Oxt is anabolic in muscle, and Oxt anti-obesogenic effects are also related to its positive effects on muscle mass. We characterized Oxt and Oxtr expression in different tissues after a cold stress (CS) challenge in mice by an in vivo approach. In this study, a cold stress protocol was elaborated and mice were kept at 4 °C for either 6 h or 5 days. Then, mice were sacrificed and properly dissected. Blood was collected and brains were quickly extracted, snap-frozen and analyzed by histomorphometry as previously shown [10]. Mice initially lost body weight, but after 5 days these mice regained their body weight, indicating that they were in good health. The exposure to cold stress activates shivering thermogenesis. In this regard, we hypothesize that there is a feedback loop between the hypothalamus and muscle that regulates the rate of central Oxt release and Oxtr expression level in the brain together with muscular Oxtr expression. This feedback loop between the brain and muscle is activated in response to situations requiring increased musculoskeletal performance such in shivering thermogenesis [11,13]. We showed in mice that Oxtr expression increases in the Soleus (Sol) muscle and in the brain after the CS challenge, while circulating Oxt decreases [10]. The dynamicity of Oxtr following a thermogenic challenge can trigger sensory deficits in ASD since Oxt regulates thermogenesis (Table 3). The cold stress model is explained in Table 3 and has been previously published [10].

### 6.2. Oxytocin Receptor Expression Is Dynamic and Is Normalized by Oxytocin During a Specific Temporal Window

The second study [14] reports that Magel2 is a gene included in the PWS locus [38], and in addition to many pathological PWS phenotypic traits, the syndrome presents a high incidence of ASD [39]. Magel2-KO mice recapitulate autistic traits and neurodevelopmental impairments. In this study, three to five hours after delivery, pups were subcutaneously injected with saline or Oxt, and this administration was repeated every 2 days. Then, 8 days after birth or 90 days after birth, mice were sacrificed, the brains were extracted and Oxtr was quantified as described [14]. An ultrasonic microphone was used to record the pups’ vocalization. The aim of this study was to extend the regional mapping of Oxtr in male and female Magel2-KO brains with or without Oxt treatment. This is important because Oxt is strongly regulated in the first week of life in mice, and understanding the specific site of action of Oxt in the brain and the specific window of time when it is effective will be essential to design a therapy with Oxt for ASD. Moreover, Oxtr is sexually dimorphic, which is why Oxtr was differently modulated according to gender after treatment with Oxt.

Early postnatal Oxt treatment prevents neonatal lethality in these mice and prevents social and learning deficits in adult Magel2-KO mice [40]. Indeed, region-specific alterations in Oxtr expression are present in Magel2-KO mice, and postnatal Oxt treatment could modulate Oxtr in specific brain regions. In Magel2-KO pups characterized by deficient production of hypothalamic Oxt [38], the neuropeptide may be unable to play its role as a mediator of early sensory functions, and the postnatal supplementation of Oxt may restore this function [14]. The action of Oxt in the brain is mediated by Oxt binding to Oxtr [39]. Environmental factors during early infancy can epigenetically modify the Oxtr gene and influence its expression level in adulthood [40]. Early modulation of Oxtr expression is important in neurodevelopmental disorders with social and cognitive deficits, as in ASD. Indeed, several mouse models of neurodevelopmental disorders present deficits in Oxt or Oxtr expression [41]. Oxtr is strongly regulated in the first three weeks of life in mice [42]. A recent study investigated whether Oxt treatment received in the first weeks of life had short- or long-term effects on regional Oxtr expression and if it is gender specific, since ASD had a higher incidence in males than in females [14]. Indeed, the Oxt system is sexually dimorphic [43]; for example, in humans, following a social stress test, a single dose of intranasal Oxt increases distress and anger in women but reduces distress in men [44]. Magel2-KO mice show highly variable region-specific patterns of Oxtr expression that vary according to Oxt administration, age and gender. Indeed, at post-natal day 8 (P8) Magel2-KO mice show a significant reduction in Oxtr levels in the brain compared to controls, indicating a major defect in Oxtr expression in the brain. This defect is present in males and females, and is not removed by the treatment with Oxt [14]. On the other hand, in Magel2-KO mice at P90, Oxt treatment normalizes Oxtr expression, specifically in those regions of the brain where Oxtr is abnormally upregulated compared to the control, such as the amygdala and hippocampus and piriform cortex, indicating an impaired developmental pattern of Oxtr expression. This effect was more evident in male mice than in female mice, which is consistent with the well-recognized sex bias in ASD incidence. One possible explanation is the existence of a temporal window in which these regions of brain are particularly sensitive to Oxt, since during neurodevelopment, brain circuits mature at different times and Oxt modulates sensory inputs and shapes brain circuits and connectivity. Conversely, when the Oxt system is defective, sensory inputs are defective or absent, failing to shape mature brain circuits. A similar time window was observed during a clinical trial with Oxt in PWS infants where Oxt administration prevents the failure to thrive of these infants only before 6 months of age [45]. The same results after Oxt administration were not detectable at any other age considered.

### 6.3. The Oxytocin System Requires Both Oxytocin/Oxytocin Receptor Expression and Synaptic Excitation/Inhibition Trasmission

The third study [46] shows that in the absence of Magel2, overall ex vivo Oxt neuron activity is suppressed for altered synaptic input profile resulting from decreased excitatory and increased inhibitory currents in mice. This shows that perturbations of the Oxt system include both neuropeptide expression [13,20] and inhibitory/excitatory synaptic transmission [46]. However, while the studies described above [13,20] rely on peptide or mRNA expression level measurement, Oxt is a signaling molecule that functions in a broader circuit of signaling and is dependent upon other hormones such as estrogens. For this reason, in a Magel2-KO mouse model, synaptic and cell autonomous properties of Oxt neurons were investigated. Magel2-KO mice were injected stereotaxically with Oxt and voltage clamp recording was performed as described [46]. Indeed, the Oxt system works through both Oxt expressing neurons and their connection and the expression of Oxt and Oxtr. The combined addition of synaptic blockers for GABAergic and glutamatergic transmission reduced the synaptic activity of Oxt neurons in control but not in Magel2-KO mice, and this indicates a loss of excitatory drive in these mice [46]. These results are consistent with previous studies on the involvement of GABA transmission in sensory deficits of ASD [2,3]. So despite the restoration of Oxt levels in ASD, Oxt neuron defects at circuit level persists, meaning that Magel2 deficiency permanently alters these circuits and the constant presence of Magel2 is essential for Oxt circuit function [37].

### 6.4. Hypothalamic Gray Matter Volume and Concentration Are Reduced in Autism Spectrum Disorder and Are Positively Associated with Peripheral Oxytocin

Finally, the fourth study [47] describes how the structural characteristics of the hypothalamus produce different results between ASD and neurotypical controls in human patients with a reduced gray matter volume or concentration in ASD children or adolescence compared with healthy controls of corresponding age. The study [47] is particularly relevant since it translates research from a mouse model to human patients. ASD individuals met DSM-5 criteria for autism and were diagnosed in accordance with current guidelines. In this study, Oxt was measured in blood samples obtained exclusively at rest to rule out any effect of Oxt on muscle contraction in ASD individuals [48]. Structural brain scans were obtained and images were processed and analyzed using voxel-based morphometry as described [47]. Notably, healthy carriers of Oxtr variants were associated with an increased likelihood of ASD, displaying a significant decrease in gray matter volume in the hypothalamus [49]. In a recent study, the morphological characteristics of the hypothalamus and their relationship with Oxt in ASD patients were analyzed through a region-of-interest analysis using voxel-based morphometry [37]. The aim of the study was to determine if there are differences in hypothalamic volume between autistic and neurotypical adults, if differences in the Oxt system in ASD were reflected in the hypothalamic structure and if these differences could be attributed to Oxt. To achieve this, the authors compared the gray matter volume between autistic and non-autistic control groups; then, they compared the differences in the relationship between hypothalamic gray matter volume and peripheral Oxt, and last, they analyzed the association between hypothalamic gray matter volume and ASD quotient scores as a measure of autistic traits [37]. Hypothalamic gray matter volume does not change between autistic and non-autistic individuals; however, comparing the group differences in terms of hypothalamic gray matter volume and peripheral Oxt, a positive association was found in the ASD group and a negative association in the non-autistic group. Hypothalamic gray matter volume was also associated with the autistic group [50]. Basal concentration of Oxt is reported to be lower in autistic children [31,48] but this difference disappears in autistic adults because of developmental changes in the Oxt system in ASD. The importance of these developmental effects has also been shown for Oxtr expression patterns [31]. This means that Oxt and Oxtr levels are dynamic and may normalize with age in ASD, translating into normalization of gray matter volume. This also means that the structural properties of the hypothalamus are related to Oxt levels in ASD [47]. This is consistent with the concept of a time window in which the treatment with Oxt is effective [51]. The most important changes in the hypothalamus and Oxt may all be modulated by variations in Oxtr expression [50]. Gray matter volume seems to increase alongside the increase in autistic traits, and this finding is probably caused by abnormalities in the Oxt system and Oxtr expression levels. These results show that the increase in Oxt levels can be caused by variations in Oxtr in the brains of ASD individuals, as seen after the cold stress challenge [13,51]. Overall, the studies described above [13,20,47,48] are consistent with the concept that Oxtr expression in the brain is dynamic and highly regulated by factors such as thermogenic challenge and muscle contraction [10], the temporal window of Oxt administration, sexual dimorphism and reproductive stage [14]. The integrity of synaptic transmission [46] and the feed-back between the brain and circulating Oxt [13,48] are also important. These data, together with the evidence that Oxt is a master gene regulating thermogenesis, made us hypothesize that the dysfunctional Oxt system could trigger sensory deficits in ASD. A role for hypothalamic hormones in sensorial functions has also been recently seen for gonadotropin-releasing hormone (GnRH) for olfactory function, consistent with our data on Oxt [52,53]. Indeed, the integrity of the Oxt system and the expression of Oxtr is essential to maintain the homeostasis of the body. This is evident, for example, in mental illnesses such as eating disorders (ED) where ED-related Oxtr haplotypes alter the relationship between proteins important for ED such as TGF-beta and sterol regulatory element-binding proteins (SREBPs) and Oxtr expression. Such disruptions cause the failure of compensatory Oxt-dependent mechanisms that would protect against starvation and stress contributing to ED [52]. Oxtr expression in the brain is directly regulated by the intake of carbohydrates and lipids [54,55], proving the dynamicity of Oxtr expression in the brain. The most recent theories explaining ASD are shown in Table 4.

## 7. Conclusions

Developmental changes can influence the structure of the hypothalamus and the expression of Oxt/Oxtr, triggering sensory deficits typically seen in autism. The action of Oxt in the brain is mediated by Oxt binding to its specific receptor Oxtr, which is a G-protein-coupled receptor expressed in several areas of the brain. An important feature of Oxtr is the variability of its distribution in the different areas of the brain and it can vary according to gender, age, pathological conditions and environmental factors [11,20]. The distribution of Oxtr in the brain varies in mammals, consistent with social behavior and sexual dimorphism. Moreover, Oxtr can undergo epigenetic modifications since early life experience has long-term effects on the Oxt system and expression of Oxtr. Early life Oxt exposure may influence life-long Oxtr expression, a theory known as “hormonal imprinting”, and it is important in neurodevelopmental disorders characterized by social abilities since many animal models of neurodevelopmental disorders present abnormalities in Oxt release and Oxtr distributions [41,44,47]. Oxt regulates thermogenesis [10,13]. Indeed, the Oxt/Oxtr system in a healthy brain is activated after thermogenic challenge, while a dysfunctional Oxt/Oxtr system in the brain may trigger sensory deficits in ASD [11,13]. In this regard, there may exist a time window for Oxt administration in ASD during which Oxt treatment is effective. Future studies are necessary to prove this preliminary evidence. It is worth noting that environmental factors may cause epigenetic modifications of the Oxtr expression in the brain, as described for thermogenic challenge, altering Oxtr expression levels in the brain, which can trigger sensory deficits in ASD. The Oxtr expression is dynamic and influenced by epigenetic factors, reproductive stage and even nutrition. This is why a limitation of this study that needs to be acknowledged is the challenge of translating animal models to human patients. Indeed, progress in the biological detection and pharmacological treatment of ASD has been limited for three main reasons. First, there were difficulties in obtaining brain-related biological samples such as brain and cerebrospinal fluid from patients with ASD. Second, the studies on ASD were performed on an animal model that lacked the sophisticated social and cognitive abilities disrupted in ASD. Third, the primate species used as a model for ASD still have several limitations [1,55,56]. We hope that the novel perspective provided in this article can add new knowledge to the understanding of ASD.

## Figures and Tables

**Table 1:** In autism spectrum disorder, sensory deficits are present in all domains of sensorial experience.

Sensory Deficits in Autism Spectrum Disorder		
Auditory	Enhanced auditory perception of details at the expense of the global situation. For example, hearing the change of a single note and not the change of the entire melody.	Hyposensitivity and hypersensitivity in all domains.
Vision	Enhanced perception of certain stimulus properties since circumscribed properties of a visual stimulus are seen over the complete picture. For example, seeing the tree and not the forest.	
Tactile	Sensorial experience changes according to the part of the body, as pressure is perceived normally on the palm of the hand and forearm, while hypersensitivity to vibrations is reported in the forearm only. Adverse response to texture is reported where vibro-tactile stimulus was generally perceived as static.	
Thermoregulation	Paradoxical heat sensation is reported where cold is perceived and burning hot and hypo- or hypersensitivity to thermal innocuous thermal stimuli is also reported according to the age of the patients. Prader–Willi syndrome individuals often present autism spectrum disorder and hypothermia and hyperpyrexia with no infection are reported.	

**Table 2:** Oxytocin and sensory deficits in autism spectrum disorder.

Oxytocin is a master gene regulating thermogenesis.
Oxytocin-deficient mice and oxytocin receptor-deficient mice have impaired thermoregulation and inability to maintain a stable temperature.
Oxytocin is released due to tactile stimuli.
Autism spectrum disorder children are reported to have a lower oxytocin concentration.
Autism spectrum disorder individuals are reported to have sensory deficits such as paradoxical heat sensations, altered thermal threshold, and hyper- or hypo-reactivity to smell, taste, audition, vision and tactile sensitivity.
The altered sensorial perception in ASD individuals can cause the cognitive and social dysfunction typical of this condition.

**Table 3:** Ex vivo study of oxytocin and oxytocin receptor expression in the brain after exposure to 6 h or 5 days of cold stress in wild-type mice and measurement of circulating oxytocin. “−” indicates a significative decrease compared to untreated control; “+” indicates a significative increase compared to untreated control. Cold stress increases Oxtr in Paraventricular and Supraoptical Nuclei of hypothalamus and decreases circulating Oxt in a feed-back/feed-forward loop with the brain [10].

**Ex vivo Studies**				
	Oxt 6 h	Oxtr 6 h	Oxt 5 d	Oxtr 5 d
Hippocampus	*	*	*	−
Hypothalamus	*	*	*	*
Paraventricular Nucleus	*	+	*	+
Supraoptical Nucleus	*	+	*	+
Circulating Oxytocin	Decreases			

* no changes detected.

**Table 4:** Most recent theories explaining autism spectrum disorder.

Theory of mind	Suggests that ASD individuals have a decreased ability to understand other people’s feelings and this can be caused by abnormalities in sensory processing.
Weak central coherence	Proposes that the meaning of things is built through the integration of information across lower-level sensory and higher-level cognitive processing and this is abnormal in ASD individuals [34].
Predictive coding hypothesis	Is based on the notion that individuals with ASD do not have a robust historical representation of the world, making it difficult for them to predict upcoming events and limiting interactions with external environments.
Reduced sensory precision and reliability	States that changes in variability of neural response pattern create variability (or less reliability) in their behaviors and perceptions.
Altered GABAergic signaling	Theory is based on evidence that the imbalance in excitatory and inhibitory processes is changed in ASD with increased excitation for greater glutamatergic signaling, while inhibition is decreased for less GABAergic signaling.
Dynamicity of oxytocin receptor expression in the brain	The variation in oxytocin receptor expression levels may cause sensory deficits, deficits in social interaction/communication and restrictive interests in autism spectrum disorder.
